# Perioperative Corticosteroid Therapy in Children Undergoing Cardiac Surgery: A Systematic Review and Meta-Analysis

**DOI:** 10.3389/fped.2020.00350

**Published:** 2020-07-24

**Authors:** Yinan Li, Qipeng Luo, Xie Wu, Yuan Jia, Fuxia Yan

**Affiliations:** Department of Anesthesiology, National Center of Cardiovascular Diseases, Fuwai Hospital, Chinese Academy of Medical Sciences and Peking Union Medical College, Beijing, China

**Keywords:** corticosteroid, children, cardiac surgery, meta-analysis, congenital heart defect

## Abstract

**Background:** The benefit–risk profile of perioperative corticosteroids in pediatric patients undergoing cardiac surgery remains controversial.

**Objective:** To investigate the influence of perioperative corticosteroids on the postoperative mortality and clinical outcomes in pediatric patients undergoing cardiac surgery with cardiopulmonary bypass.

**Methods:** We conducted a systematic search using MEDLINE, EMBASE, and Cochrane Database through August 31, 2019. We included randomized controlled trials comparing perioperative corticosteroids with other clinical interventions, placebo, or no treatment in children between 0 and 18 years of age undergoing cardiac surgery. The primary outcome of interest was all-cause in-hospital mortality. The secondary outcomes were length of intensive care unit stay (LOIS), duration of mechanical ventilation (DMV), postoperative insulin therapy, postoperative low cardiac output syndrome (LCOS), postoperative infection, maximal temperature (*T*_max_) in the first 24 h postoperatively, urine output (UO) in the first 24 h postoperatively, serum lactate at postoperative day (POD) 1, blood glucose at POD 1, vasoactive inotrope score (VIS) at POD 1, and postoperative acute kidney injury (AKI). Study quality was assessed using the Cochrane Risk of Bias Assessment Tool.

**Results:** Our analysis included 17 studies and 848 pediatric patients. The data demonstrated that children receiving corticosteroids showed no significant difference on the all-cause in-hospital mortality with a fixed-effect model (RR = 0.59, 95% CI = 0.28–1.25, *P* = 0.55) compared with controls. For the secondary outcomes, corticosteroids had a statistically significant reduction on the VIS at POD1 (MD = −2.04, 95% CI = −3.96 −0.12, *P* = 0.04), while it might be significantly associated with an increased blood glucose at POD1 (MD = 1.38, 95% CI = 0.68–2.09, *P* = 0.0001) and a 2.69-fold higher risk of postoperative insulin therapy (RR = 2.69, 95% CI = 1.37–5.27, *P* = 0.004). No statistical significance was shown in other secondary outcomes.

**Conclusion:** Perioperative corticosteroids might not significantly improve clinical outcomes identified as mortality, LOIS, DMV, AKI, and LCOS other than VIS at POD1. However, it might increase the blood glucose and episodes of insulin therapy. Perioperative corticosteroids to attenuate the inflammatory response are not supported by available evidence from our study. Further results from ongoing randomized controlled trials with a larger sample size are required.

## Introduction

It is necessary to be exposed to cardiopulmonary bypass (CPB) for most pediatric patients undergoing repair or palliative congenital heart surgery. CPB may contribute to a complex pathophysiology process as a consequence of exposure to the artificial surface of CPB circuits, hemodilution, hypothermia, ischemia/reperfusion of important organs, etc., on which excessive inflammatory system activation characterized by the release of proinflammatory cytokines and aggregation of neutrophils plays an important role (1, 2). Despite the improved perfusion strategies and other agents to alleviate the inflammatory response during CPB, corticosteroids have been used for almost half a century as the first-line agent with remaining controversies in this field (3).

Corticosteroids can relieve inflammatory response by reducing endotoxin and proinflammatory cytokine release (4). In addition, they may directly act as a supplementary therapy for adrenal insufficiency as a result of CPB in neonates and infants (5). However, some potential adverse effects may be associated with corticosteroids, for example, hyperglycemia, poor wound healing, infections, and poor neurodevelopmental outcomes (6–8). Several small, randomized, controlled studies in pediatric patients have investigated the effect of perioperative corticosteroids on the inflammatory biomarkers, clinical outcomes, and adverse events after congenital heart surgeries (9–25). Nevertheless, the results were conflicted, resulting in the controversy over perioperative corticosteroids. Due to the result of a recent retrospective study, which demonstrated increased morbidities associated with perioperative corticosteroids in lower-risk patients instead of benefits for clinical outcomes, we should be alerted and reexamine the risk/benefit ratio of perioperative corticosteroids.

Therefore, it is necessary to systematically review the existing studies so as to assess the beneficial and adverse effects of perioperative corticosteroids, which may furthermore guide the decision-making on perioperative corticosteroids in pediatric cardiac surgery.

We performed a systematic review of previous literatures and conducted a meta-analysis restricted to randomized controlled trials (RCTs) to determine the effects of perioperative corticosteroids on outcomes in patients undergoing pediatric cardiac surgery.

## Methods

### Search Strategy

Medline, Embase, and Cochrane Database were searched for articles published since inception until August 31, 2019, with a publication language restricted to English only. Two investigators (LYN, LQP) independently conducted the search and examined the bibliographies to identify potential related articles ready to be recruited. The searching strategies were as follows: [(cardiac surgery OR valve surgery OR coronary surgery) OR (cardiopulmonary bypass OR extracorporeal circulation)] AND (glucocorticoid OR steroid OR hydrocortisone OR dexamethasone OR methylprednisolone) AND (infant OR congenital OR pediatric OR pediatric OR children OR neonatal).

### Eligibility Criteria

The two investigators referred in the searching process (LYN, LQP) screened titles and abstracts of the retrieved publications in accordance with the following inclusion criteria: (i) published in English; (ii) carried out as randomized controlled clinical trials; (iii) age of patients included in the original studies below 18 years; (iv) scheduled for congenital cardiac surgery; and (v) steroid perioperatively administered. Studies without reporting the primary or secondary outcomes or with all outcomes being reported as median and interquartile range were excluded from the study.

### Data Extraction and Outcome Measures

This review was conducted following the instruction of the Cochrane Handbook for Systematic Reviews of Intervention (Version 6, 2019) (26). A predesigned data collection form was used to extract data from original studies (Table S1). Two investigators (LYN, LQP) independently perform the data extraction, and a third investigator (WX) would involve if there was any discrepancy between the data extracted from the same study. The primary outcome was all-cause in-hospital mortality. The secondary outcomes included (i) length of intensive care units stay (LOIS); (ii) duration of mechanical ventilation (DMV); (iii) postoperative insulin therapy; (iv) postoperative low cardiac output syndrome (LCOS); (v) postoperative infection; (vi) maximal temperature (*T*_max_) in the first 24 h postoperatively; (vii) urine output (UO) in the first 24 h postoperatively; (viii) serum lactate at postoperative day (POD) 1; (ix) blood glucose at POD1; (x) vasoactive inotrope score (VIS) at POD1; and (xi) postoperative acute kidney injury (AKI). Additional information extracted included gender, age, weight, type of surgery, study country, study period, sample size, type of corticosteroids, administration regimen, and therapy for the control group.

### Risk of Bias Assessment

**Figure 1:**
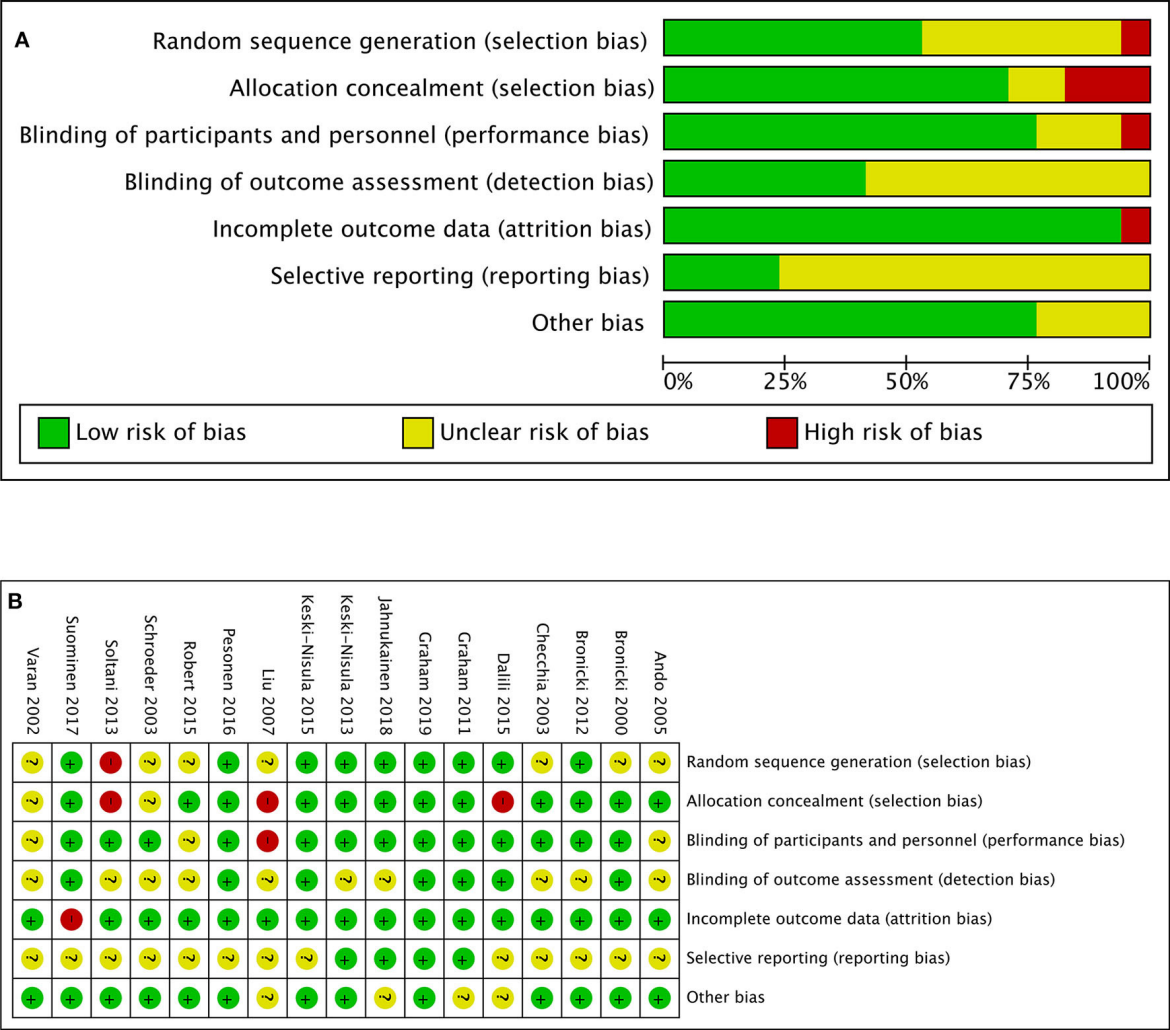
**(A)** Risk-of-bias graph: review authors' judgments about each risk-of-bias item presented as percentages across all included studies. **(B)** Risk-of-bias summary: review authors' judgments about each risk-of-bias item for each included study.

The “risk of bias” table integrated in the Review Manager (REVMAN) software (version 5.3; The Nordic Cochrane Center, Copenhagen, Denmark) was used to perform the quality assessment of studies included in the meta-analysis, which consisted of the parameters of bias such as random sequence generation, allocation concealment, blinding of participants and personnel, blinding of outcome assessment, incomplete outcome data, selective reporting, and other bias. Each parameter was graded as “low,” “high,” or “uncertain” to classify its risk of bias (Figure 1). The same two investigators (LYN, LQP) finished the assessment independently, with a third investigator (WX) resolving the discrepancy.

### Statistical Analysis

All analysis was performed in Review Manager (REVMAN) software (version 5.3; The Nordic Cochrane Center, Copenhagen, Denmark). The relative risks (RRs) or weighted mean differences (WMDs) and their corresponding 95% confidence intervals (95% CIs) were calculated for dichotomous and continuous outcomes' data, respectively.

Both the chi-squared and I-squared statistics were used to assess study heterogeneity. I-squared values of 25, 50, and 75% were defined as low, median, and high heterogeneity thresholds, respectively, and *p* < 0.1 were considered indicative of heterogeneity.

The fixed-effects model was used predominantly in all analysis. However, a random-effects model was employed if significant heterogeneity was found among the included studies. A funnel plot was employed to assess publication bias. *P* < 0.05 were considered statistically significant.

## Results

**Figure 2:**
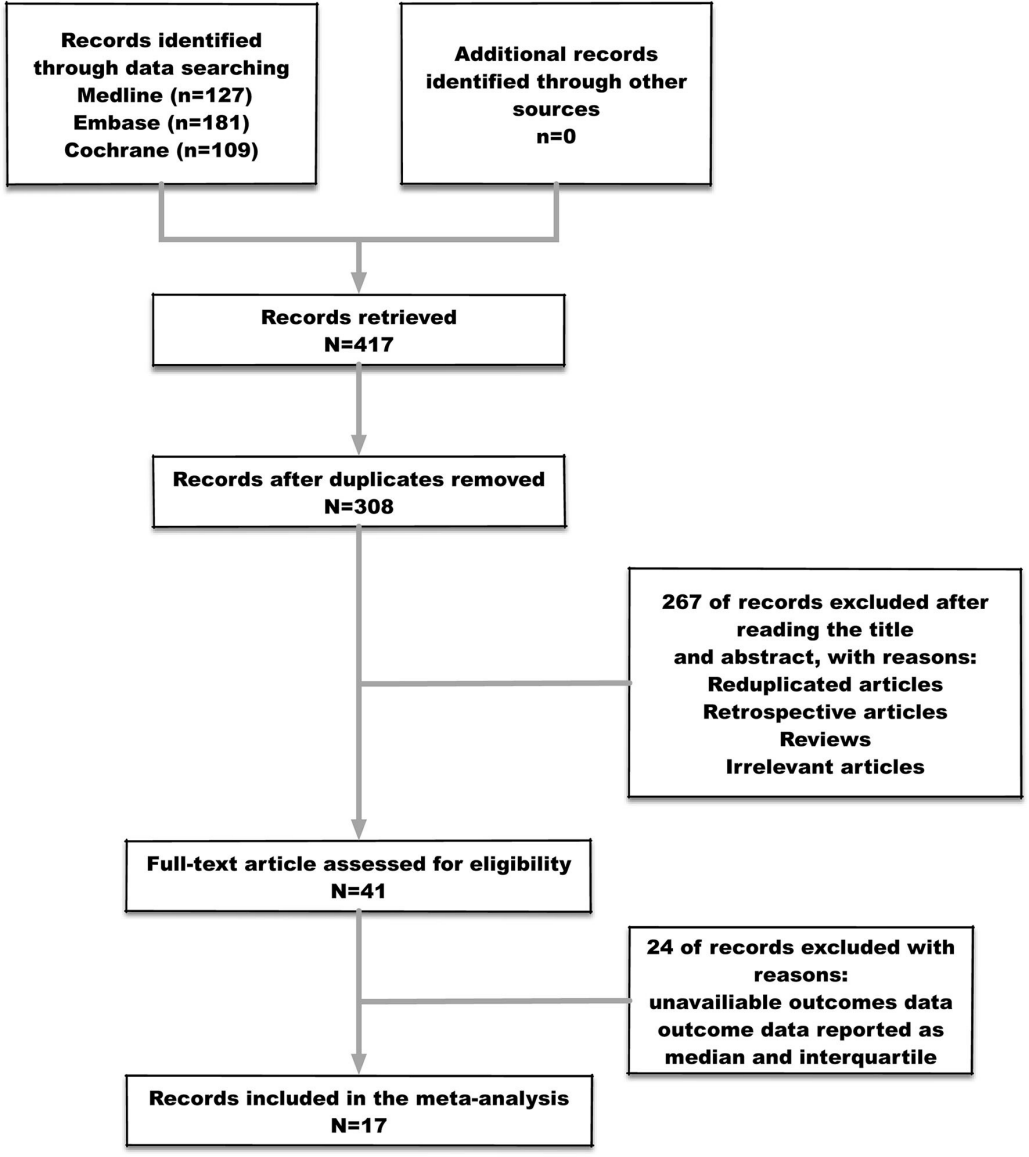
Flow diagram of the study selection process.

Four hundred seventeen studies were identified after the initial searching, among which 109 were duplicates and consequently removed from the study. Three hundred eight studies were screened for the titles and abstracts, after which 267 studies were excluded for reasons including reduplicated articles, retrospective articles, review articles, and irrelevant articles. Subsequently, full texts of 41 articles were assessed for inclusion. Eventually, 17 studies (Table 1) met the predefined criteria and were included in the final meta-analysis. The process of studies' screening and selection was described in Figure 2.

**Table 1:** Basic data of 17 included trials.

**References**	**Type of RCT**	**Groups**		**Participants**	**Drugs**	**Dosing regimen**	**Control**	**Endpoints**
		**T**	**C**					
Ando et al. (13)	Single center	10	10	Neonates undergoing complete biventricular repair	Hydrocortisone	0.18 mg/kg/h for 3 d, 0.09 mg/kg/h for 2 d, and 0.045 mg/kg/h for 2 d postoperatively	Placebo	DMV, LOIS, blood glucose at POD1, VIS at POD1
Bronicki et al. (9)	Single center	15	14	Children undergoing open heart surgical procedures for congenital heart defects.	Dexamethasone	1 mg/kg, maximum dose of 10 mg/kg, immediately after induction of anesthesia	Placebo	Mortality, AKI
Bronicki et al. (16)	Single center	18	13	Children who required CPB to correct structural heart disease	Dexamethasone	1 mg/kg, 8 h before CPB and after induction of anesthesia	Placebo	DMV, LOIS
Checchia et al. (11)	Single center	15	13	Children who underwent open-heart surgery for congenital heart defects	Dexamethasone	1 mg/kg, 1 h before initiation of CPB	Placebo	Mortality
Dalili et al. (19)	Single center	50	50	Children undergoing total repair of Fallot's tetralogy	Methylprednisolone	30 mg/kg, after admission to ICU	Placebo	DMV, LOIS, LCOS, postoperative infection
Graham et al. (15)	Single center	39	37	Neonates (aged 30 days) who were scheduled to undergo cardiac surgery involving CPB	Methylprednisolone	30 mg/kg, after induction of anesthesia	Placebo	Mortality, DMV, LOIS, postoperative insulin therapy, postoperative infection
Graham et al. (25)	Multicenter	81	95	Infants ≤ 1 month (31 days) of age scheduled to undergo cardiac surgery with CPB	Methylprednisolone	30 mg/kg, after induction of anesthesia	Placebo	Mortality, postoperative insulin therapy, LCOS, AKI
Jahnukainen et al. (24)	Single center	20	20	Neonates (age ≤ 28 days) who were undergoing elective open-heart surgery with CPB due to congenital heart defects	Methylprednisolone, hydrocortisone	2 mg/kg, after induction of anesthesia; 0.2 mg/kg/h, 6 h postoperatively	Placebo	AKI
Keski-Nisula et al. (8)	Single center	20	20	Neonates (age 28 days or younger) undergoing open-heart surgery	Methylprednisolone	30 mg/kg, after induction of anesthesia	Placebo	Mortality, DMV, LOIS, blood glucose at POD1, postoperative insulin therapy, VIS at POD1, *T*_max_ in the first 24 h postoperatively, serum lactate st POD1
Keski-Nisula et al. (20)	Single center	15	15	Children between 1 and 18 months of age undergoing VSD or AVSD repair	Methylprednisolone	30 mg/kg, after induction of anesthesia	Placebo	DMV, LOIS, blood glucose at POD1, VIS at POD1, *T*_max_ in the first 24 h postoperatively, serum lactate st POD1
Liu et al. (14)	Single center, single blinded	15	15	Infants with congenital heart disease undergoing cardiac surgery with CPB	Methylprednisolone	30 mg/kg, in the priming solution of CPB	Zero-balance ultrafiltration	DMV, LOIS
Pesonen et al. (22)	Single center	15	15	Children between 1 and 18 months of age undergoing VSD or AVSD repair	Methylprednisolone	30 mg/kg, after induction of anesthesia	Placebo	UO in the first 24 h postoperatively
Robert et al. (21)	Single center	19	21	Infants undergoing surgical repairs typically performed in the neonatal period that required CPB	Hydrocortisone	50 mg/m^2^, in the operating room after removal of CPB; 50 mg/m^2^/d, began immediately after the loading dose; continued for 48 h and then tapered over 3 days as follows: 40 mg/m^2^/d × 24 h, 30 mg/m2/d × 12 h, 20 mg/m^2^/d × 12 h, 10 mg/m^2^/d × 24 h, then stop.	Placebo	Mortality, LCOS, postoperative infection, AKI
Schroeder et al. (12)	Single center	14	15	Children undergoing CPB for repair of congenital heart disease	Methylprednisolone	30 mg/kg, 4 h before CPB	Placebo	DMV, LOIS, VIS at POD1, LCOS, *T*_max_ in the first 24 h postoperatively, postoperative infection, UO in the first 24 h postoperatively
Abbasi Tashnizi et al. (17)	Single center, single blinded	39	40	Children younger than 5 years who underwent CPB for repair of congenital heart disease	Methylprednisolone	30 mg/kg, 4 h before CPB	Placebo	
Suominen et al. (23)	Single center	20	20	Neonates (age 28 days) who were undergoing nonemergency cardiac operations with CPB	Methylprednisolone, hydrocortisone	2 mg/kg, after induction; starting from 6 h after the weaning from the CPB at the rate of 0.2 mg/kg/h for 48 h, 0.1 mg/kg/h for 48 h, and 0.05 mg/kg/h for 24 hours.	Placebo	Mortality, DMV, LOIS, blood glucose at POD1, postoperative insulin therapy, postoperative infection, serum lactate st POD1
Varan et al. (10)	Single center	15	15	Children undergoing cardiac surgery with CPB	Methylprednisolone	30 mg/kg, before onset of CPB	Methylprednisolone, 2 mg/kg before onset of CPB	DMV, LOIS, *T*_max_ in the first 24 h postoperatively, UO in the first 24 h postoperatively

*AKI, acute kidney injury; CPB, cardiopulmonary bypass; DMV, duration of mechanical ventilation; LCOS, low cardiac output syndrome; LOIS, length of intensive care unit stay; POD, postoperative day; **T**_**max**_, maximal temperature; UO, urine output; VIS, vasoactive inotrope score*.

### Mortality, Duration of Mechanical Ventilation, and Length of Intensive Care Unit Stay

**Figure 3:**
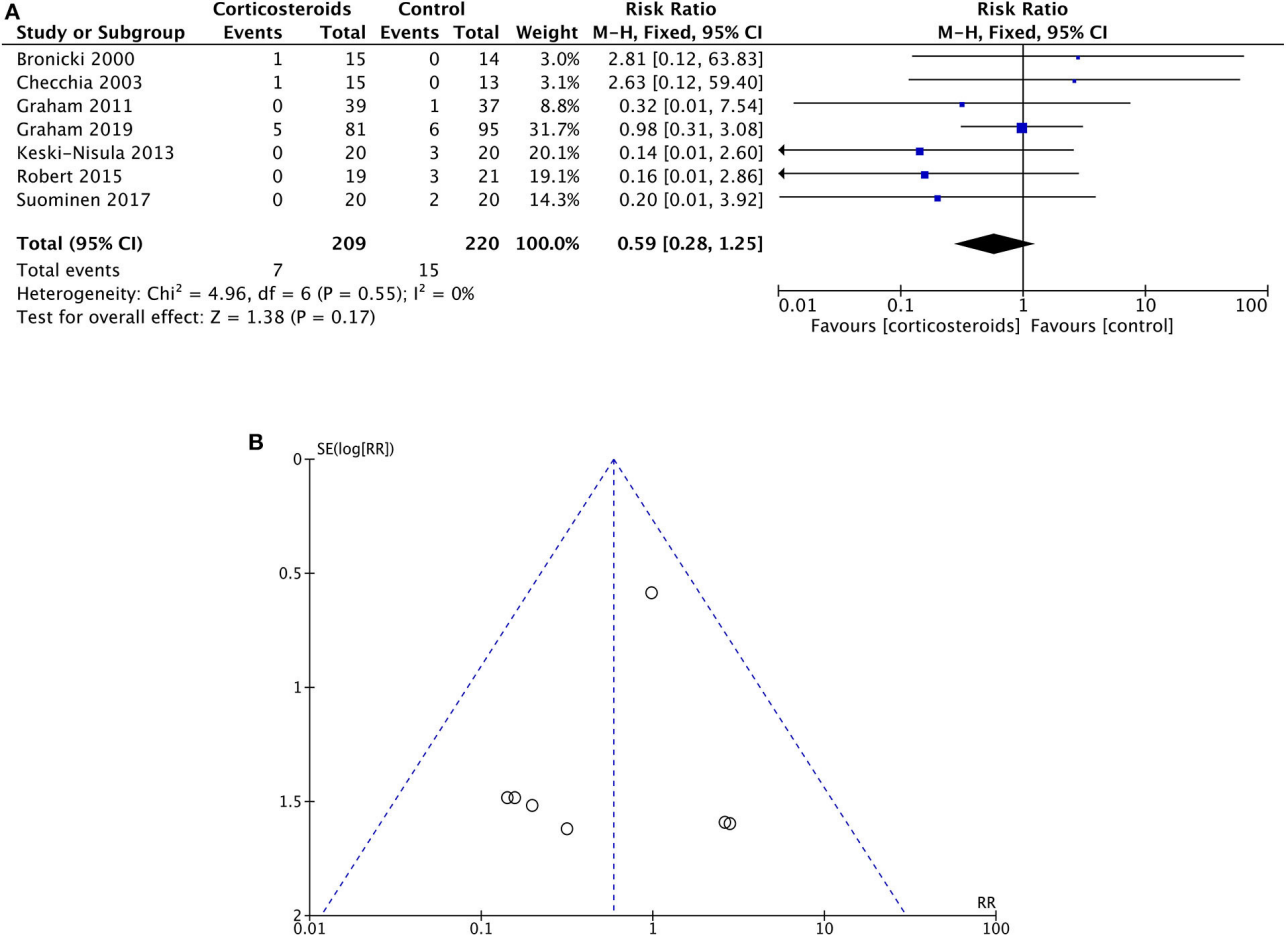
**(A)** Forest plot of corticosteroid treatment vs. control on mortality. **(B)** Funnel plot of corticosteroid treatment vs. control on mortality.

Four hundred twenty-nine patients from seven studies were assessed for the postoperative all-cause in-hospital mortality after surgeries, of which 209 patients received corticosteroid therapy while others did not. The mortalities in the corticosteroid therapy group and the control group were 3.3 and 6.8%, respectively, without any significant difference being detected between groups (RR: 0.59; 95% CI (0.28, 1.25); *P* = 0.17) (Figure 3). Heterogeneity was low among studies with Chi^2^ = 4.69, *P* = 0.55, and with *I*^2^ = 0%. However, one study (25) almost had half of the included population. As a result, the sensitivity analysis was performed to detect the effect of each study in outcomes. Consequently, removing any single study did not influence the final result on mortality.

**Figure 4:**
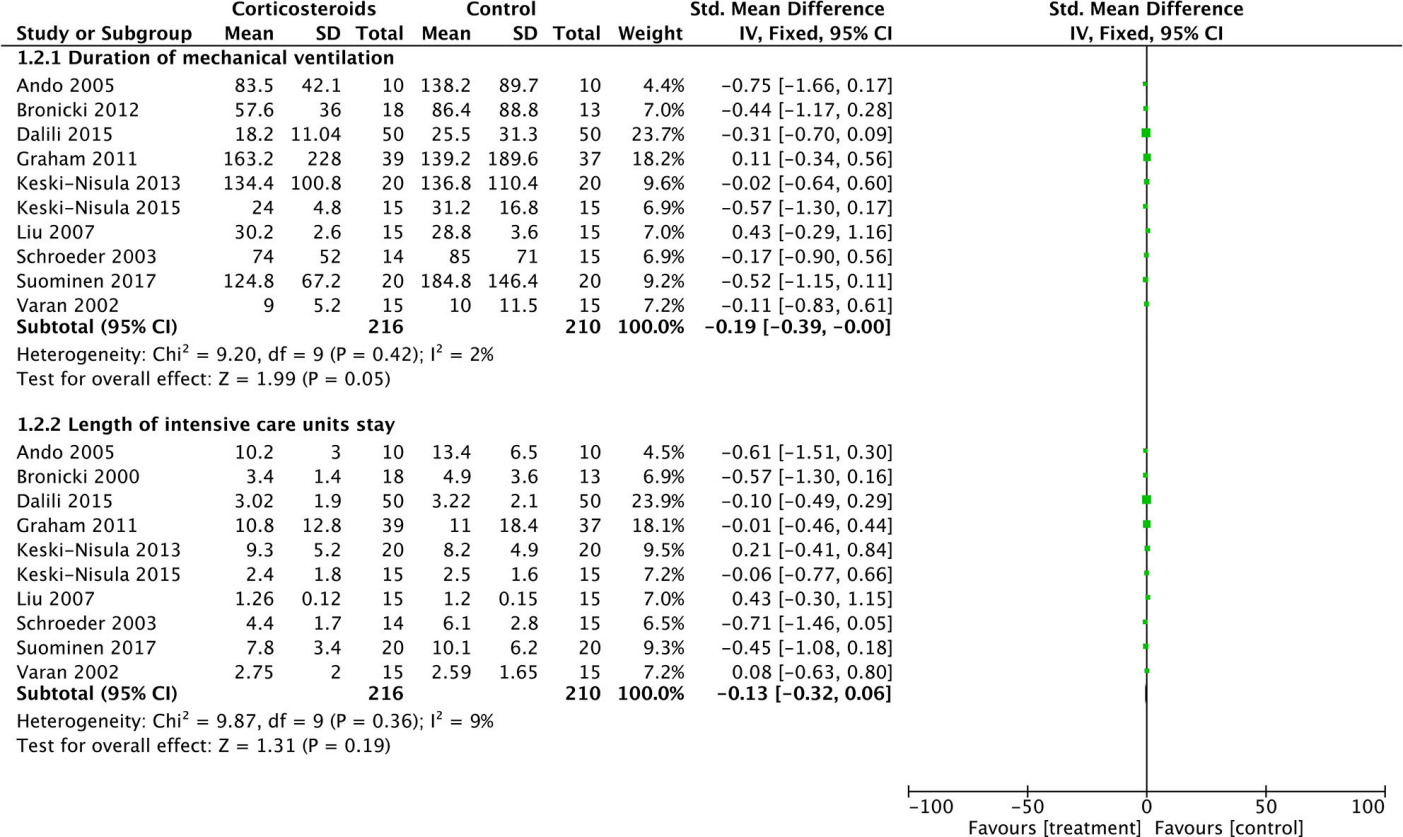
Forest plot of corticosteroid treatment vs. control on duration of mechanical ventilation and length of intensive care unit stay.

As for DMV and LOIS analysis, 416 patients from 10 studies were eventually analyzed. Corticosteroid therapy did not significantly decrease DMV (SMD: −0.19; 95% CI (−0.39, 0); *P* = 0.05) and LOIS (SMD: −0.13; 95% CI (−0.32, 0.06); *P* = 0.19) (Figure 4). There was no significant difference in heterogeneity identified between studies with *Chi*^2^ = 9.87, *P* = 0.36, *I*^2^ = 9%, and *Chi*^2^ = 9.2, *P* = 0.42, *I*^2^ = 2%, respectively.

### Blood Glucose and Insulin Therapy

**Figure 5:**
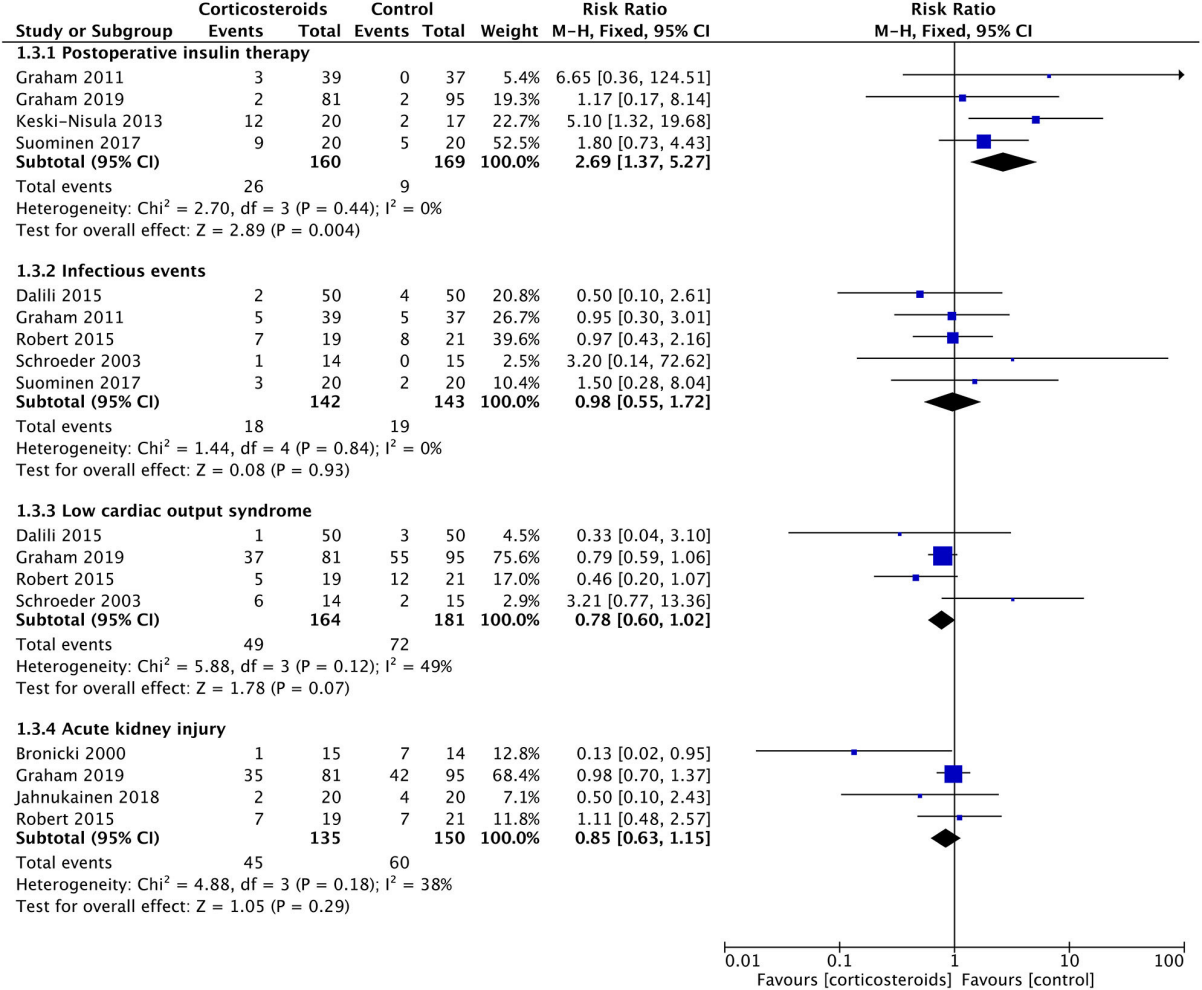
Forest plot of corticosteroid treatment vs. control on postoperative insulin therapy, infectious events, low cardiac output syndrome, and acute kidney injury.

**Figure 6:**
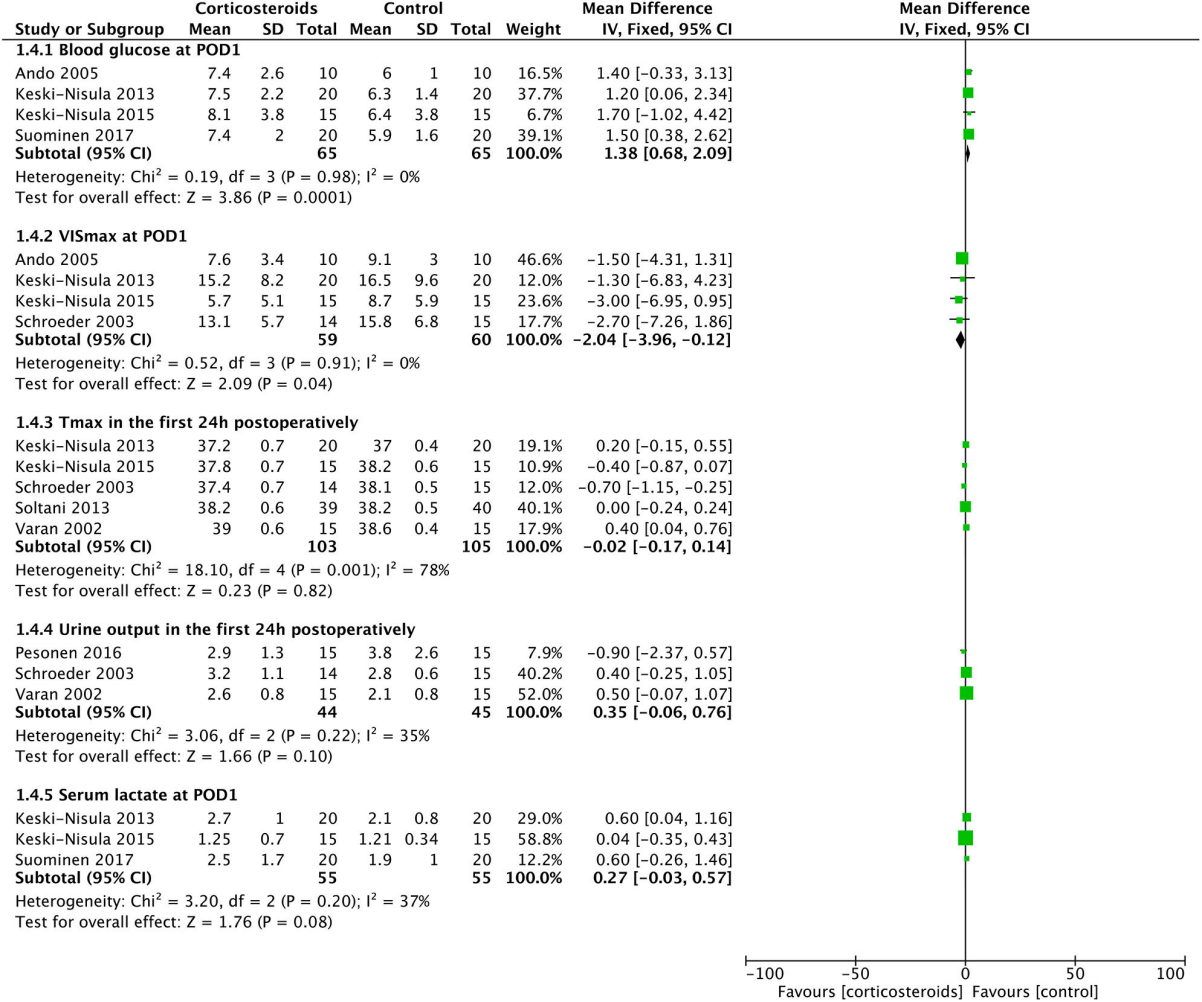
Forest plot of corticosteroid treatment vs. control on blood glucose at POD1, VISmax at POD1, maximal temperature in the first 24 h postoperatively, urine output in the first 24 h postoperatively, and serum lactate at POD1.

Corticosteroid therapy significantly increased the blood glucose at POD1 (MD: 1.38; 95% CI (0.68, 2.09); *P* = 0.0001) (**Figure 6**). There was no significant heterogeneity existing between studies (*Chi*^2^ = 0.19, *P* = 0.98, and with *I*^2^ = 0%) (**Figure 6**). Meanwhile, corticosteroids were significantly associated with a 2.69-fold higher risk of postoperative insulin therapy (16.3% vs. 5.3%; RR: 2.69; 95% CI (1.37, 5.27); *P* = 0.004) without effect of heterogeneity between studies (*Chi*^2^ = 2.70, *P* = 0.44, and with *I*^2^ = 0%) (Figure 5).

### Vasoactive Inotrope Score at Postoperative Day 1 and Low Cardiac Output Syndrome

Corticosteroid therapy had a significantly favorable reduction on the VIS at POD1 compared with controls (MD: −2.04; 95% CI (−3.96, −0.12); *P* = 0.04) (Figure 6). However, the incidence of LCOS showed no significant difference between groups with or without corticosteroid therapy (RR, 0.78; 95% CI (0.60, 1.02); *P* = 0.07) (Figure 5). These two results were not influenced by heterogeneity between studies (*Chi*^2^ = 0.52, *P* = 0.91, and with *I*^2^ = 0% in the former; *Chi*^2^ = 5.88, *P* = 0.12, and with *I*^2^ = 49% in the latter).

### Maximal Temperature in the First 24 h Postoperatively and Postoperative Infection

*T*_max_ in the first 24 h postoperatively was similar in the two groups (MD: −0.08; 95% CI (−0.42, 0.27); *P* = 0.66) (Figure 6). The results were derived from a random-effects model as a result of the high heterogeneity between studies (*Chi*^2^ = 18.1, *P* = 0.01, *I*^2^ = 78%). The rates of postoperative infection events were also without any significant difference (12.7% vs. 13.3%; RR, 0.98; 95% CI (0.55, 1.72); *P* = 0.93) (Figure 5). The result was not influenced by heterogeneities between studies (*Chi*^2^ = 1.44, *P* = 0.84, *I*^2^ = 0%).

### Urine Output in the First 24 h Postoperatively and Acute Kidney Injury

Three studies with 85 patients reported the UO in the first 24 h postoperatively, with no significant difference detected between the corticosteroid group and the controlled group (MD: 0.35; 95% CI (−0.06, 0.76); *P* = 0.10) (Figure 6). Furthermore, the risks of acute kidney injury were similar between groups (33.3% vs. 40%; RR, 0.85; 95% CI (0.63, 1.15); *P* = 0.29) (Figure 5). Non-significantly low heterogeneity was demonstrated between studies, which had no effect on the results (Chi^2^ = 4.88, *P* = 0.18, and with *I*^2^ = 38% in the former; Chi^2^ = 3.06, *P* = 0.22, and with *I*^2^ = 35% in the latter).

### Serum Lactate at Postoperative Day 1

Three studies with 110 patients reported the serum lactate level at POD1. No significant difference was identified between the corticosteroid group and the controlled group (MD: 0.27; 95% CI (−0.03, 0.57); *P* = 0.08), which was not influenced by the non-significantly low heterogeneity between studies (Chi^2^ = 3.20, *P* = 0.20, *I*^2^ = 37%) (Figure 6).

## Discussion

Seventeen randomized controlled studies compared the effects of perioperative corticosteroids with controls on clinical outcomes in pediatric patients undergoing congenital cardiac surgeries. The included studies use different corticosteroids consisting of dexamethasone, methylprednisolone, and hydrocortisone in different doses within the time range between 4 h before CPB initiation and several days postoperatively. Despite of the variability among different studies, several important findings emerged.

For primary outcomes, perioperative corticosteroids may not reduce the postoperative all-cause in-hospital mortality, DMV, and LOIS. Systemic inflammatory response syndrome (SIRS) was common in patients undergoing CPB as a result of contacting with extracorporeal circuits, endothelial cell infiltration, and ischemia–reperfusion injury (27). Children were regarded as the most vulnerable population to this kind of SIRS due to more severe hemodilution, small circulating volume, and the implementation of more complex surgery procedures than adults (28). Corticosteroids have always been the soundest and most controversial therapy agents to inhibit the SIRS related to CPB. However, the pathophysiologic procedures as a consequence of CPB referred above were relieved significantly with the development of perfusion strategy and postoperative management strategy, resulting in decrease in mortality and morbidity (29). Early extubation became more common in some institutions either (30). Therefore, whether or to which degree the direct inhibition of SIRS by corticosteroids would eventually convert into better clinical outcomes was miserable.

A multicenter retrospective study including 46,370 children undergoing cardiac surgeries demonstrated that perioperative corticosteroids were not related to the decrease in mortality and DMV while they might significantly prolong LOIS (31). The similar result was found in another retrospective research involving neonatal patients (32). A recent randomized controlled study of 176 patients also failed to show a significant difference between corticosteroids and placebo in mortality, DMV, and LOIS with the analysis of two institutions adopting somewhat different perioperative management strategies (25), while the benefit of intraoperative corticosteroids was significant in one institution.

A meta-analysis (33) formulated with six studies demonstrated no significant improvement in clinical outcomes other than renal function with perioperative corticosteroids in neonates, while there seemed to be a trend toward a reduction in mortality of the corticosteroid group. As a consequence of the different types, regimens, and timings of corticosteroids in the involved studies of the meta-analysis, the reliability of the meta-analysis was at a lower level. In a study (34) including a more specialized population undergoing high-risk cardiac surgeries with an Aristotle score of 10 or greater, intraoperative corticosteroids shortened LOIS and achieved a further reduction in DMV with an additional preoperative dose. It seemed that perioperative corticosteroids are not as effective for clinical outcomes as for inflammatory biomarkers, especially in a low-risk population.

More supplementary results from the ongoing multicenter trials Steroids to Reduce Systemic Inflammation after Neonatal Heart Surgery (NCT03229538) and Dexamethasone in pEdiatric Cardiac Surgery (NCT02615262) might contribute to define the proper population in which the perioperative corticosteroid might be beneficial for the improvement on clinical outcomes.

For secondary outcomes, perioperative corticosteroids were associated with an elevated blood glucose value and more episodes of postoperative insulin therapy. The included studies for the assessment of blood glucose value and episodes of postoperative insulin therapy were totally different. Therefore, it was improper to distinguish whether the elevated blood glucose was the reason for postoperative insulin therapy, while hyperglycemia could be considered as the most common reason in the study population. It was reported that hyperglycemia was associated with poor clinical outcomes such as prolonged LOIS, more infectious episodes, and even increased mortality in severe patients. However, the infection episodes and maximal temperature in the first 24 h postoperatively were similar between groups as well as LOIS and mortality. We must admit that the studies included enrolled patients with different conditions, especially for the three (10, 17, 20) of them, in which only patients undergoing simple congenital heart surgeries were enrolled. These three studies played important roles on assessing the infection episodes and maximal temperature in the first 24 h postoperatively, which might be the reason for the high heterogeneity between studies in the assessment above. Consequently, whether the elevated blood glucose would influence on the final outcomes of patients was unclear.

AKI occurs in 5–33% patients undergoing congenital cardiac surgeries with CPB, on which the inflammatory response plays an important role. However, our meta-analysis demonstrated no difference between corticosteroids and controls in AKI. Morgan et al. (35) demonstrated that despite of the reduction in proinflammatory cytokines due to intraoperative corticosteroid use, it was not independently associated with AKI after cardiac surgery in children. It might imply that the mechanism of pediatric cardiac surgery-associated AKI was beyond the scope of just excessive inflammatory response. The *post hoc* analysis of severe AKI in the Dexamethasone for Cardiac Surgery (DECS) trial found that intraoperative high-dose dexamethasone could only reduce AKI in high-risk adults with advanced chronic kidney dysfunction (36), which indicated the limited effect of corticosteroid on AKI after the surgery technique, perfusion, and anesthesia strategies were all improved. Modified ultrafiltration used in pediatric cardiac surgeries to remove the excessive water and inflammatory mediators during CPB not only reduced the need for transfusions but also improved the associated organ injury. In addition, three (21, 24, 25) of the studies included to assess the effect of corticosteroids on AKI were conducted within the recent 5 years. Some patients in their studies received circuit primes without blood products. Meanwhile, minimized circuits were applied to some of the neonatal patients, which decreased the exposing surface directly, lowering the inflammation-associated AKI. In this circumstance, it was likely that the effect of corticosteroids would be too tiny to be covered.

Several studies demonstrated that corticosteroids might decrease the serum myocardial enzyme and improve cardiac function after congenital cardiac surgeries. Our analysis showed that corticosteroid therapy could significantly reduce the vasoactive agents used at POD1 with a tendency toward a lower rate of LCOS. Because of the heterogeneity between studies, more randomized studies should be carried out to identify whether it was just a tendency or a difference.

## Study Limitations

There were some limitations in our study. First, we included studies using various doses, timings, and types of corticosteroids, which indicated for a subgroup analysis if enough subjects were enrolled. Second, children undergoing cardiac surgeries at all age ranges were included in this study. However, as we all know, neonates were of great difference in physiology from elder children, which reduced the generalization of the results. Meanwhile, three studies (10, 17, 20) we included only enrolled patients undergoing simple congenital heart surgeries with shorter CPB duration, which might complicate the results. Third, the 30-mg/kg dose of methylprednisolone was too much for clinical practice in some institutions. The risk–benefit profile was not applicable for these institutions. Fourth, most of the included studies had a small sample size, which may result in a small-study effect.

## Conclusions

We demonstrated that perioperative corticosteroids in pediatric patients undergoing cardiac surgeries might not significantly improve clinical outcomes identified as LOIS, DMV, mortality, AKI, and LCOS other than vasoactive agents' usage at POD1. However, perioperative corticosteroids might increase the blood glucose level and episodes of insulin therapy. Perioperative corticosteroids to attenuate the inflammatory response are not supported by the available evidence from our study. More results from the ongoing randomized controlled trials Steroids to Reduce Systemic Inflammation after Neonatal Heart Surgery (NCT03229538) and Dexamethasone in pEdiatric Cardiac Surgery (NCT02615262) were urgently necessary to conclude the risk–benefit profile of perioperative corticosteroids.

## Data Availability Statement

The raw data supporting the conclusions of this article will be made available by the authors, without undue reservation.

## Author Contributions

FY, YJ, and YL contributed to the conception and design of the study. YL, QL, and XW screened the title and abstract, selected the studies, assessed the quality of evidence, extracted the data, and performed the analysis. FY and YJ supervised the study selection and data analysis. YL drafted the initial manuscript. All authors contributed to the manuscript revision and read and approved the submitted version.

## Conflict of Interest

The authors declare that the research was conducted in the absence of any commercial or financial relationships that could be construed as a potential conflict of interest.

## References

[1] Tarnok A Emmrich F. Immune consequences of pediatric and adult cardiovascular surgery: report of the 7th Leipzig workshop. Cytometry Part B Clin Cytom. (2003) 54:54–7. 10.1002/cyto.b.10018 12827668

[2] Levy JH Tanaka KA. Inflammatory response to cardiopulmonary bypass. Ann Thorac Surg. (2003) 75:S715–20. 10.1016/S0003-4975(02)04701-X 12607717

[3] Replogle RL Gazzaniga AB Gross RE. Use of corticosteroids during cardiopulmonary bypass: possible lysosome stabilization. Circulation. (1966) 33(4 Suppl.):I86–92. 10.1161/01.CIR.33.4S1.I-86 5933603

[4] Hall RI Smith MS Rocker G. The systemic inflammatory response to cardiopulmonary bypass: pathophysiological, therapeutic, and pharmacological considerations. Anesth Analg. (1997) 85:766–82. 10.1097/00000539-199710000-00011 9322454

[5] Graham EM Bradley SM. First nights, the adrenal axis, and steroids. J Thorac Cardiovasc Surg. (2017) 153:1164–6. 10.1016/j.jtcvs.2016.12.013 28131511

[6] Elhoff JJ Chowdhury SM Zyblewski SC Atz AM Bradley SM Graham EM. Intraoperative steroid use and outcomes following the norwood procedure: an analysis of the pediatric heart network's public database. Pediatr Crit Care med. (2016) 17:30–5. 10.1097/PCC.0000000000000541 26492058 PMC4703451

[7] Sprung CL Annane D Keh D Moreno R Singer M Freivogel K. Hydrocortisone therapy for patients with septic shock. N Engl J Med. (2008) 358:111–24. 10.1056/NEJMoa071366 18184957

[8] Yeh TF Lin YJ Lin HC Huang CC Hsieh WS Lin CH. Outcomes at school age after postnatal dexamethasone therapy for lung disease of prematurity. N Engl J Med. (2004) 350:1304–13. 10.1056/NEJMoa032089 15044641

[9] Bronicki RA Backer CL Baden HP Mavroudis C Crawford SE Green TP. Dexamethasone reduces the inflammatory response to cardiopulmonary bypass in children. Ann Thorac Surg. (2000) 69:1490–5. 10.1016/S0003-4975(00)01082-1 10881828

[10] Varan B Tokel K Mercan S Donmez A Aslamaci S. Systemic inflammatory response related to cardiopulmonary bypass and its modification by methyl prednisolone: high dose vs. low dose. Pediatr Cardiol. (2002) 23:437–41. 10.1007/s00246-002-0118-3 12170362

[11] Checchia PA Backer CL Bronicki RA Baden HP Crawford SE Green TP. Dexamethasone reduces postoperative troponin levels in children undergoing cardiopulmonary bypass. Crit Care Med. (2003) 31:1742–5. 10.1097/01.CCM.0000063443.32874.60 12794414

[12] Schroeder VA Pearl JM Schwartz SM Shanley TP Manning PB Nelson DP. Combined steroid treatment for congenital heart surgery improves oxygen delivery and reduces postbypass inflammatory mediator expression. Circulation. (2003) 107:2823–8. 10.1161/01.CIR.0000070955.55636.25 12756159

[13] Ando M Park IS Wada N Takahashi Y. Steroid supplementation: a legitimate pharmacotherapy after neonatal open heart surgery. Ann Thorac Surg. (2005) 80:1672–8. Discussion: 8. 10.1016/j.athoracsur.2005.04.035 16242437

[14] Liu J Ji B Long C Li C Feng Z. Comparative effectiveness of methylprednisolone and zero-balance ultrafiltration on inflammatory response after pediatric cardiopulmonary bypass. Artif Organs. (2007) 31:571–5. 10.1111/j.1525-1594.2007.00423.x 17584482

[15] Graham EM Atz AM Butts RJ Baker NL Zyblewski SC Deardorff RL. Standardized preoperative corticosteroid treatment in neonates undergoing cardiac surgery: results from a randomized trial. J Thorac Cardiovasc Surg. (2011) 142:1523–9. 10.1016/j.jtcvs.2011.04.019 21600592 PMC3161127

[16] Bronicki RA Checchia PA Stuart-Killion RB Dixon DJ Backer CL. The effects of multiple doses of glucocorticoids on the inflammatory response to cardiopulmonary bypass in children. World J Pediatr Congenit Heart Surg. (2012) 3:439–45. 10.1177/2150135112447544 23804905

[17] Abbasi Tashnizi M Soltani G Moeinipour AA Ayatollahi H Tanha AS Jarahi L. Comparison between preoperative administration of methylprednisolone with its administration before and during congenital heart surgery on serum levels of IL-6 and IL-10. Iran Red Crescent Med J. (2013) 15:147–51. 10.5812/ircmj.8039 24349746 PMC3840835

[18] Keski-Nisula J Pesonen E Olkkola KT Peltola K Neuvonen PJ Tuominen N. Methylprednisolone in neonatal cardiac surgery: reduced inflammation without improved clinical outcome. Ann Thorac Surg. (2013) 95:2126–32. 10.1016/j.athoracsur.2013.02.013 23602068

[19] Dalili M Vesal A Tabib A Khani-Tafti L Hosseini S Totonchi Z. Single dose corticosteroid therapy after surgical repair of Fallot's tetralogy; a randomized controlled clinical trial. Res Cardiovasc Med. (2015) 4:e25500. 10.5812/cardiovascmed.25500 25789260 PMC4350157

[20] Keski-Nisula J Suominen PK Olkkola KT Peltola K Neuvonen PJ Tynkkynen P. Effect of timing and route of methylprednisolone administration during pediatric cardiac surgical procedures. Ann Thorac Surg. (2015) 99:180–5. 10.1016/j.athoracsur.2014.08.042 25440273

[21] Robert SM Borasino S Dabal RJ Cleveland DC Hock KM Alten JA. Postoperative hydrocortisone infusion reduces the prevalence of low cardiac output syndrome after neonatal cardiopulmonary bypass. Pediatr Crit Care Med. (2015) 16:629–36. 10.1097/PCC.0000000000000426 25901540

[22] Pesonen EJ Suominen PK Keski-Nisula J Mattila IP Rautiainen P Jahnukainen T. The effect of methylprednisolone on plasma concentrations of neutrophil gelatinase-associated lipocalin in pediatric heart surgery. Pediatr Crit Care Med. (2016) 17:121–7. 10.1097/PCC.0000000000000573 26509817

[23] Suominen PK Keski-Nisula J Ojala T Rautiainen P Jahnukainen T Hastbacka J. Stress-Dose corticosteroid vs. placebo in neonatal cardiac operations: a randomized controlled trial. Ann Thorac Surg. (2017) 104:1378–85. 10.1016/j.athoracsur.2017.01.111 28434547

[24] Jahnukainen T Keski-Nisula J Tainio J Valkonen H Patila T Jalanko H. Efficacy of corticosteroids in prevention of acute kidney injury in neonates undergoing cardiac surgery-A randomized controlled trial. Acta Anaesthesiol Scand. (2018) 62:13134. 10.1111/aas.13134 29667173

[25] Graham EM Martin RH Buckley JR Zyblewski SC Kavarana MN Bradley SM. Corticosteroid therapy in neonates undergoing cardiopulmonary bypass: randomized controlled trial. J Am Coll Cardiol. (2019) 74:659–68. 10.1016/j.jacc.2019.05.060 31370958 PMC6684326

[26] Julian Higgins JT Jacqueline Chandler Miranda Cumpston Tianjing Li Matthew Page Vivian Welch Cochrane Handbook for Systematic Reviews of Interventions. Available online at: https://training.cochrane.org/handbook/current Cochrane 2019 (accessed July, 2019).

[27] Laffey JG Boylan JF Cheng DC. The systemic inflammatory response to cardiac surgery: implications for the anesthesiologist. Anesthesiology. (2002) 97:215–52. 10.1097/00000542-200207000-00030 12131125

[28] Anand KJ Hansen DD Hickey PR. Hormonal-metabolic stress responses in neonates undergoing cardiac surgery. Anesthesiology. (1990) 73:661–70. 10.1097/00000542-199010000-00012 2221435

[29] Jacobs ML O'Brien SM Jacobs JP Mavroudis C Lacour-Gayet F Pasquali SK. An empirically based tool for analyzing morbidity associated with operations for congenital heart disease. J Thorac Cardiovasc Surg. (2013) 145:1046–57.e1. 10.1016/j.jtcvs.2012.06.029 22835225 PMC3824389

[30] Mahle WT Nicolson SC Hollenbeck-Pringle D Gaies MG Witte MK Lee EK. Utilizing a collaborative learning model to promote early Extubation following infant heart surgery. Pediatr Crit Care Med. (2016) 17:939–47. 10.1097/PCC.0000000000000918 27513600 PMC5053873

[31] Pasquali SK Hall M Li JS Peterson ED Jaggers J Lodge AJ. Corticosteroids and outcome in children undergoing congenital heart surgery: analysis of the pediatric health information systems database. Circulation. (2010) 122:2123–30. 10.1161/CIRCULATIONAHA.110.948737 21060075 PMC3013053

[32] Pasquali SK Li JS He X Jacobs ML O'Brien SM Hall M. Perioperative methylprednisolone and outcome in neonates undergoing heart surgery. Pediatrics. (2012) 129:e385–91. 10.1542/peds.2011-2034 22271697 PMC3269116

[33] Scrascia G Rotunno C Guida P Amorese L Polieri D Codazzi D. Perioperative steroids administration in pediatric cardiac surgery: a meta-analysis of randomized controlled trials^*^. Pediatr Crit Care Med. (2014) 15:435–42. 10.1097/PCC.0000000000000128 24717907

[34] Clarizia NA Manlhiot C Schwartz SM Sivarajan VB Maratta R Holtby HM. Improved outcomes associated with intraoperative steroid use in high-risk pediatric cardiac surgery. Ann Thorac Surg. (2011) 91:1222–7. 10.1016/j.athoracsur.2010.11.005 21440149

[35] Morgan CJ Gill PJ Lam S Joffe AR Peri-operative interventions, but not inflammatory mediators, increase risk of acute kidney injury after cardiac surgery: a prospective cohort study. Intensive Care Med. (2013) 39:934–41. 10.1007/s00134-013-2849-4 23417202

[36] Jacob KA Leaf DE Dieleman JM van D Nierich AP Rosseel PM. Intraoperative high-dose dexamethasone and severe AKI after cardiac surgery. J Am Soc Nephrol. (2015) 26:2947–51. 10.1681/ASN.2014080840 25952257 PMC4657835

