# Supplementary material for: Perioperative Corticosteroid Therapy in Children Undergoing Cardiac Surgery: A Systematic Review and Meta-Analysis
Source: Front Pediatr. 2020 Jul 24;8:350. doi: 10.3389/fped.2020.00350 (PMC7396528; doi:10.3389/fped.2020.00350)
Supplement: Supplementary file 1 [file Table_1.DOCX]

**Table S1: A data abstraction form to collect the trial design and outcomes data from the original studies.**

| **Questions** | **Answers** | |
| --- | --- | --- |
| **AUTHORS** |  | |
| **PUBLICATION ID** |  | |
| **YEAR OF PUBLICATION** |  | |
| **LANGUAGE** |  | |
| **TYPE OF STUDY** |  | |
| **COMMENTS ON STUDY DESIGN** | | |
| **Allocation was concealed and drawn consecutively** |  | |
| **Disclosure of Allocation was Possible** |  | |
| **Allocation concealment was not stated or was unclear** |  | |
| **Allocation was not concealed (e.g. quasi ‐randomisation)** |  | |
| **Inclusion and exclusion criteria were clearly defined in the text?** |  | |
| **Inclusion and exclusion criteria were not clearly defined in the text?** |  | |
| **Outcomes of patients who withdrew or were excluded from after allocation were**  **EITHER detailed separately or included in an intention to treat analysis or the text stated that there were no withdrawals.** |  | |
| **Outcome of patients who withdrew or were excluded after allocation were**  **NEITHER detailed separately nor included in an intention to treat analysis** |  | |
| **Treatment and control groups were adequately described at entry**  **(a minimum of 4 admission details were described (age, sex, weight, allergies))** |  | |
| **Treatment and control groups were NOT adequately described at entry** |  | |
| **The text stated that the care programmes other than the trial options were identical** |  | |
| **The text stated that the care programmes other than the trial options were NOT identical** |  | |
| **Outcome measures were clearly defined in the text** |  | |
| **Outcome measures were NOT clearly defined in the text** |  | |
| **Outcome assessors were blind to the allocation of patients** |  | |
| **Outcome assessors were NOT blind to the allocation of patients** |  | |
| **The timing of outcome measures was appropriate** |  | |
| **The timing of outcome measures was NOT appropriate** |  | |
| **METHODS** |  | |
| **Physician‐ Blinded** |  | |
| **Outcome assessor blinded** |  | |
| **PARTICIPANTS** |  | |
| **Number of eligible participants** |  | |
| **Number enrolled in study** |  | |
| **Number of treatment group** |  | |
| **Number of control group** |  | |
| **Number Males : Females** |  | |
| **Age range** |  | |
| **Type of surgery** |  | |
| **Were groups similar at entry** |  | |
| **INTERVENTION** |  | |
| **Type of control group** |  | |
| **Steroid type** |  | |
| **Dose** |  | |
| **Timing** |  | |
| **Withdrawals** |  | |
| **Drop outs** |  | |
| **Included in analysis** |  | |
| **COMMENT ON TREATMENT** | **TREATMENT GROUP** | **CONTROL GROUP** |
| **Mortality (n(%))** |  |  |
| **Duration of ventilation (h)** |  |  |
| **Length of intensive care units stay (d)** |  |  |
| **Postoperative acute kidney injury (n(%) )** |  |  |
| **Urine output in the first 24h postoperatively (mL/kg/h)** |  |  |
| **Postoperative insulin therapy (n (%))** |  |  |
| **Blood glucose at postoperative day 1 (mmol/L)** |  |  |
| **Postoperative infection (n (%))** |  |  |
| **Maximal temperature in the first 24h postoperatively (℃)** |  |  |
| **Low cardiac output syndrome (n (%))** |  |  |
| **Vasoactive inotrope score at postoperative day 1** |  |  |
| **Serum lactate (mmol/L)** |  |  |
| **Authors contacted regarding unreported outcome data**  **Date contacted Response received** |  |  |
| **COMMENTS ON OUTCOMES** |  |  |
